# Calomel and Tartar Emetic Stricken from the Supply Table of the Army

**Published:** 1863-06

**Authors:** W. A. Hammond

**Affiliations:** Surgeon Gen’l.; Surgeon-General’s Office, Washington, D. C.


Calomel and Tartar Emetic Stricken from the Supply Table of the Army.—I. From the reports of Medical Inspectors and the Sanitary reports to this office, it appears that the administration of calomel has so frequently been pushed to excess by military surgeons as to call for prompt steps by this office to correct this abuse; an abuse the melancholy effects of which, as officially reported, have exhibited themselves not only in innumerable cases of profuse salivation, but in the not unfrequent occurrence of mercurial gangrene.

It seeming impossible in any other manner to properly restrict the use of this powerful agent, it is directed that it be struck from the supply table, and that no further requisitions for this medicine be approved by Medical Directors. This is done with the more confidence, as modern pathology has proved the impropriety of the use of mercury in very many of those diseases in which it was formerly unfailingly administered.

II. The records of this office having conclusively proved that diseases prevalent in the army may be treated as efficiently without tartar emetic as therewith, and the fact of its remaining upon the supply table being a tacit invitation to its use, tartar emetic is also struck from the supply table of the army.

No doubt can exist that more harm has resulted from the misuse of both these agents, in the treatment of disease, than benefit from their proper administration. W. A. Hammond, Surgeon Gen'l.

Surgeon-General's Office, Washington, D. C.,
May 4th, 1863.

